# MicroRNAs predict early complications of autologous hematopoietic stem cell transplantation

**DOI:** 10.1186/s40364-024-00585-x

**Published:** 2024-04-23

**Authors:** Damian Mikulski, Mateusz Nowicki, Izabela Dróżdż, Ewelina Perdas, Piotr Strzałka, Kacper Kościelny, Małgorzata Misiewicz, Konrad Stawiski, Agnieszka Wierzbowska, Wojciech Fendler

**Affiliations:** 1 Department of Biostatistics and Translational Medicine, Medical University of Lodz, Lodz, Poland; 2 Department of Hematology, Medical University of Lodz, Lodz, Poland; 3 Department of Hematology and Transplantology, Provincial Multi-Specialized Oncology and Trauma Center, Lodz, Poland; 4 Department of Clinical Genetics, Medical University of Lodz, Lodz, Poland; 5 Department of Radiation Oncology, Dana-Farber Cancer Institute, Boston, MA USA

**Keywords:** miRNA, Autologous hematopoietic stem cell transplant, Hsa-miR-223-3p, Hsa-miR-15b-5p, Hsa-miR-126-5p, Bacteremia, Biomarkers

## Abstract

Autologous hematopoietic stem cell transplantation (AHSCT) remains the most prevalent type of stem cell transplantation. In our study, we investigated the changes in circulating miRNAs in AHSCT recipients and their potential to predict early procedure-related complications. We collected serum samples from 77 patients, including 54 with multiple myeloma, at four key time points: before AHSCT, on the day of transplantation (day 0), and at days + 7 and + 14 post-transplantation. Through serum miRNA-seq analysis, we identified altered expression patterns and miRNAs associated with the AHSCT procedure. Validation using qPCR confirmed deviations in the levels of miRNAs at the beginning of the procedure in patients who subsequently developed bacteremia: hsa-miR-223-3p and hsa-miR-15b-5p exhibited decreased expression, while hsa-miR-126-5p had increased level. Then, a neural network model was constructed to use miRNA levels for the prediction of bacteremia. The model achieved an accuracy of 93.33% (95%CI: 68.05-99.83%), with a sensitivity of 100% (95%CI: 67.81-100.00%) and specificity of 90.91% (95%CI: 58.72-99.77%) in predicting bacteremia with mean of 6.5 ± 3.2 days before occurrence. In addition, we showed unique patterns of miRNA expression in patients experiencing platelet engraftment delay which involved the downregulation of hsa-let-7f-5p and upregulation of hsa-miR-96-5p; and neutrophil engraftment delay which was associated with decreased levels of hsa-miR-125a-5p and hsa-miR-15b-5p. Our findings highlight the significant alterations in serum miRNA levels during AHSCT and suggest the clinical utility of miRNA expression patterns as potential biomarkers that could be harnessed to improve patient outcomes, particularly by predicting the risk of bacteremia during AHSCT.

**Supplementary Information:**

The online version contains supplementary material available at 10.1186/s40364-024-00585-x.

**To the editor**

Autologous hematopoietic stem cell transplantation (AHSCT) is broadly used to treat hematologic disorders (predominantly multiple myeloma), with an estimated 28,700 procedures performed in Europe in 2019 [1]. Attempts to establish AHSCT as an outpatient procedure are gaining traction, but concerns about adverse effects like mucositis, bacteremia or delayed engraftment (DE) limit this transition [2, 3]. Conventional cytokine or cell count-based biomarkers may be unreliable in predicting or detecting those complications in AHSCT recipients due to the nature of the procedure itself. In the present study, we aimed to quantify alterations in the signature of freely circulating miRNAs in the sera of AHSCT recipients and identify circulating miRNAs that could be used to create a predictive model for bacteremia - a common and potentially life-threatening complication of AHSCT [4–6].

Serum samples were taken from all patients (*N* = 77; Table 1 and Supplementary Table 1) at four time points throughout AHSCT. miRNA-seq was performed to identify potential miRNA biomarkers (N1 = 10). MiRNAs with profiles affected by AHSCT were subsequently validated with a targeted qPCR (N2 = 67) for their association with bacteremia and other complications. The detailed Methods were presented in Supplementary File 1 and Supplementary Fig. 1.

**Table 1:** Clinical characteristics of patients included into the study

Variable	*N* = 77 (100%)		
Disease	MM: 54 (70.1) HL: 11 (14.3) MCL: 5 (6.5) DLBCL: 3 (3.9) Other: 4 (5.2)		
Conditioning regimen	Mel-200: 39 (50.6) Reduced Mel: 16 (20.8) BeEAM: 17 (22.1) BEAM: 5 (6.5)		
Variable	MM	ML	*p*
Sex	F: 29 (53.7) M: 25 (46.3)	F: 11 (47.8) M: 12 (52.2)	0.8233
Age at AHSCT Median (25–75%), years	61.3 (53.8–66.4)	47.4 (39.5–54.1)	< 0.0001
Number of CD34 + cells transplanted (× 10^6^ cells/kg) Median (25–75%)	3.9 (3.1–5.3)	4.6 (3.3–5.7)	0.4092
Mucositis- any grade	34 (64.2)	23 (100.0)	0.0004
Mucositis– grade ≥ 2	18 (34.0)	22 (95.7)	< 0.0001
Bacteremia	11 (20.4)	8 (34.8)	0.2920
Neutropenic fever	30 (58.8)	18 (78.3)	0.1745
Days to PLT engraftment Median (25–75%)	10 (10–13)	15 (12–18)	0.0001
Days to ANC engraftment Median (25–75%)	11	10	0.0299

ANC- absolute neutrophil count; BEAM- carmustine, etoposide, cytarabine, melphalan; BeEAM- bendamustine, etoposide, cytarabine, melphalan; DLBCL- diffuse large B-cell lymphoma; HL- Hodgkin Lymphoma; 25–75%- interquartile range; MCL- mantle cell lymphoma; MEL- melphalan; ML- malignant lymphoma; MM- multiple myeloma; PLT- platelets

In miRNA-seq data, dysregulation of miRNAs expression across study time points was shown with 20 miRNAs showing a significant difference in global repeated measures ANOVA (Fig. 1A, Supplementary File 2). Twelve miRNAs were identified as eligible for qPCR validation (Fig. 1B and H and Supplementary Figs. 2 and 3) due to their significant fluctuations across the procedure and association with DE. Additional five miRNAs associated with irrevocable bone marrow damage (miR-150-5p, miR-375, miR-122-5p, hsa-miR-126-5p, and miR-122b-3p) and four potential reference miRNAs, two of which (hsa-miR-27b-3p and hsa-miR-148b-3p) were the final normalization factor to provide controls and calibration [7–9].

**Fig. 1:**
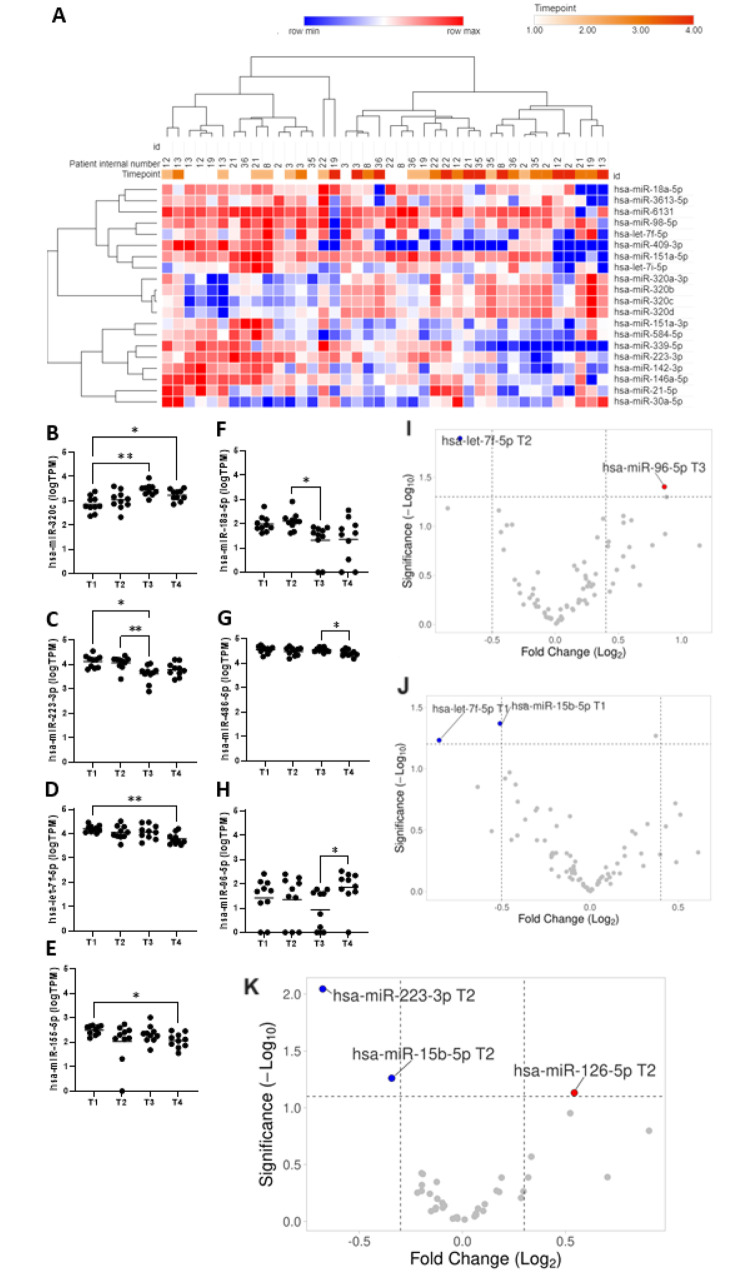
miRNA-seq analysis results. Samples were drawn at four timepoints: (T1) before conditioning chemotherapy, (T2) on the day of AHSCT (day 0), day + 7 (T3), and + 14 day after AHSCT (T4). (**A**) heatmap of miRNAs differently expressed across study timepoint assessed by repeated measures ANOVA. The serum miRNA profiles tend to cluster by the study time points- two clusters- “early” (T1 and T2) and “late” (T3 and T4) are visible. One minus Pearson correlation distance metric and complete linkage method were used. (**B-H**) Plots for seven miRNAs differentially expressed across AHSCT procedure in miRNA-seq stage of the study. There were no statistically significant results in the comparison of miRNAs expression between at T1 and T2. In T3, hsa-miR-320c (**B**) was significantly upregulated compared to T1 (FC = 3.92, *p* = 0.007), whereas hsa-miR-223-3p (**C**) was significantly downregulated (FC 0.31, *p* = 0.048). MiRNA levels at T4, in comparison to T1, showed significant downregulation of both hsa-let-7f-5p (**D**) and hsa-miR-155-5p (**E**) (FC = 0.38, *p* = 0.004 and FC = 0.37, *p* = 0.019, respectively), while hsa-miR-320c (**B**) was significantly upregulated (FC = 2.39, *p* = 0.049). At T3, there was lower expression of hsa-miR-18a-5p (**F**) (FC = 0.16, *p* = 0.035) and hsa-miR-223-3p (**C**) (FC = 0.35, *p* = 0.033) comparing to T2. Comparing T4 with T3, a lower expression level of hsa-miR-486-5p (**G**) (FC = 0.72, *p* = 0.024) was identified. In a comparison of T4 with T2, a higher expression level of hsa-miR-96-5p (**H**) (FC = 8.33, *p* = 0.036) was observed. Asterisks denote the significance level (paired t-test with Bonferroni correction): *- *p* ≤ 0.05; **- *p* ≤ 0.01. (**I-J**) Volcano plots showing differentially expressed miRNAs in patients with platelet delayed engraftment (DE) (**I**) and neutrophil DE (**J**). Red dots represent upregulated miRNAs; blue dots represent downregulated miRNAs; grey dots represent miRNAs with no significant difference. (**K**) Volcano plot showing differentially expressed miRNAs in patients who developed bacteremia. Only miRNAs in T1 and T2 (before the event occurrence) were included in the analysis to establish potential predictors for further classifier development

Overall, the results of both methods- miRNA-seq and RT-qPCR were highly convergent across all time points (Supplementary Fig. 4). All five miRNAs related to radiotherapy-induced response changed their expression significantly across the study time points (Supplementary Fig. 5) confirming their association with bone marrow damage.

In the RT-qPCR group, thirteen patients had neutrophil DE with lower expression of hsa-miR-125a-5p (FC_T3_=0.77, *p* = 0.0301) and hsa-miR-15b-5p (FC_T1_=0.70, *p* = 0.0428); while 13 had platelet DE time which was associated with hsa-let-7f-5p (FC_T2_=0.59, *p* = 0.0128) and hsa-miR-96-5p (FC_T3_=1.82, *p* = 0.0397) levels (Fig. 1I-J, Supplementary File 3).

In total, there were 17 episodes of documented bacteremia in the RT-qPCR set of patients. The majority were caused by Gram-positive bacteria (13, 76.5%). The mean time to bacteremia onset since AHSCT (T2) was 6.5 ± 3.2 days. Using miRNA levels at baseline or T2 a neural network (NN) model (Supplementary Files 4–7) for bacteremia prediction was iteratively designed. The final model relied on three miRNAs quantified at T2 were included: hsa-miR-223-3p, hsa-miR-15b-5p, and hsa-miR-126-5p (Fig. 1K, Supplementary Fig. 6) and showed accuracy of 93.33%, 95%CI:68.05-99.83% (Supplementary File 6) in the validation group with one false positive case occurred in the validation set (sensitivity 100%; specificity 90.91%, 95%CI: 58.72-99.77%). With the prevalence of bacteremia in the entire studied group, the positive predictive value reached 94.12% (95%CI: 69.61-99.11%) while NPV equaled 98.00% (95%CI: 87.97-99.70%).

Pathway analysis using the KEGG database demonstrated that miRNAs retained in the model were enriched for genes associated with various infections and responses to infections, including Hepatitis C, Toxoplasmosis, Salmonella infection, Shigellosis, Influenza A, Measles, Herpes simplex infection, Bacterial invasion of epithelial cells and Fc gamma R-mediated phagocytosis (Supplementary Figs. 7 and 8). Moreover, all three miRNAs included in our model were identified and predicted to originate from potential tissue sources that are predominantly affected by the AHSCT procedure (Supplementary Fig. 9).

Our study is the first to assess circulating miRNA expression patterns during AHSCT and identify biomarkers of the procedure’s complications. Notably, we observed expression changes in relation to complications such as bacteremia and engraftment delay. Interestingly, the differentially expressed miRNAs largely manifested prior to the onset of these complications. Our findings culminated in developing a predictive model distinguishing patients at risk of developing bacteremia– a critical and life-threatening AHSCT complication [4].

While we strove to include a balance of different indications for AHSCT, the relatively small sample size may have resulted in a bias toward the variable selection of miRNAs associated with particular underlying diseases. Replicable patterns of miRNAs identified earlier as associated with bone marrow damage seem to show that severe stimuli exert expression changes that are evident despite baseline differences [7, 10]. The evidence suggests that the individual miRNAs integrated into our model have also been independently associated with sepsis and severe infections across diverse patient cohorts. Specifically, hsa-miR-223-3p, hsa-miR-15b-5p, and hsa-miR-126-5p have consistently demonstrated connections to these events, irrespective of the underlying hematologic diagnoses [11, 12]. Nevertheless, independent external validation would strengthen their clinical relevance. Our findings regarding the association of circulating miRNA expression patterns incurred by bone marrow damage could extend beyond the setting of AHSCT, aiding targeted interventions to mitigate myelotoxicity and enhance the safety of other cancer treatments or detection of exposure to myelotoxic stimuli.

The underlying prior data on miRNA biomarkers of myelotoxicity concerned the Total Body Irradiation (TBI) procedure [7, 8]. In those patients - with different malignancies, clinical factors, and procedures- the impact of miRNAs was clearly evidenced and maintained regardless of clinical confounding factors. We thus hypothesize that the myeloablative procedure is an event of such catastrophic impact on the organism level that it overshadows other causes of miRNA expression variability at the serum level. Deregulated miRNAs consistently changed post high versus low radiation doses, with hsa-miR-150-5p, hsa-miR-122-5p, hsa-miR-122b-3p decreasing, and hsa-miR-375, hsa-miR-126-5p increasing after radiotherapy [7]. In the current study, hsa-miR-150-5p declined, while hsa-miR-375 and hsa-miR-126-5p were over-expressed across AHSCT, mirroring changes during TBI.

In conclusion, our study shows distinct patterns of miRNA in chemotherapy-induced injury across AHSCT which may be used to predict bacteremia and potentially stratifying patients as eligible for outpatient AHSCT.

### Electronic supplementary material

Below is the link to the electronic supplementary material.

Supplementary Material 1

Supplementary Material 2

Supplementary Material 3

Supplementary Material 4

Supplementary Material 5

Supplementary Material 6

Supplementary Material 7

## Data Availability

All data generated and analyzed during this study are included in this published article and its supplementary files.
