# Supplementary material for: MicroRNAs predict early complications of autologous hematopoietic stem cell transplantation
Source: Biomark Res. 2024 Apr 23;12:42. doi: 10.1186/s40364-024-00585-x (PMC11036737; doi:10.1186/s40364-024-00585-x)
Supplement: Supplementary file 1 — Supplementary Material 1 [file 40364_2024_585_MOESM1_ESM.docx]

**Table of contents:**

***- file uploaded separately**

**Supplementary File 1**. Detailed Patients and Methods section. Includes:

**Supplementary Table 1.** Clinical characteristics of patients included into the study.

**Supplementary Figure 1**. The graphical representation of the study protocol.

**Supplementary Figure 2.** miRNA expression from miRNA-seq stage of the study- volcano plots demonstrating pairwise comparisons between study timepoints. A. Paired comparison- T1 vs T2, none of miRNAs remained statistically significant after false discovery rate (FDR) correction. B. T1 vs T3- four miRNAs were significantly upregulated- hsa-miR-320b (fold change, FC=3.1, p=0.007); hsa-miR-320c (FC= 3.9, p=0.007), hsa-miR-320a-3p (FC=2.0, p=0.009) and hsa-miR-320d (FC=3.9, p=0.042) and one miRNA was significantly downregulated- hsa-miR-223-3p (FC=0.3, p=0.048). C. T1 vs T4- three miRNAs were significantly downregulated: hsa-let-7f-5p (FC=0.4, p=0.004), hsa-let-7i-5p (FC=0.6, p=0.010), hsa-miR-155-5p (FC=0.4, p=0.019); one miRNA was significantly upregulated- hsa-miR-320c (FC= 2.4, p=0.049). D. T2 vs T3- two miRNAs were significantly downregulated- hsa-miR-18a-5p (FC=0.2, p=0.035) and hsa-miR-223-3p (FC=0.4, p=0.033). E. T3 vs T4- two miRNAs were significantly deregulated- hsa-miR-486-5p (FC=0.7, p=0.024) and hsa-miR-96-5p (FC=8.3, p=0.036). F. T2 vs T4- none of miRNAs remained statistically significant after FDR correction.

**Supplementary Figure 3.** Correlations between time to engraftment and expression level of miRNAs measured at baseline (timepoint 1) in the miRNA-seq stage of the study. In total 5 miRNAs that showed significant correlations with both neutrophil and platelet engraftment times were subsequently selected for qPCR validation. (A, B) hsa-miR-125a-5p; (C, D) hsa-miR-99b-5p; (E, F) hsa-miR-361-3p; (G, H) hsa-miR-486-3p; (I, J) hsa-miR-92b-3p.

**Supplementary Figure 4.** Validation of sequencing data using RT-qPCR. Comparisons of log2FC values between miRNA-seq and RT-qPCR in subsequent timepoints: A. T1 vs T2; B. T1 vs T3; C. T1 vs. T4.

**Supplementary Figure 5.** Changes in serum expression of miRNAs related to radiotherapy response. (A) hsa-miR-150-5p had the highest expression at T1, with the drop after administration of conditioning chemotherapy (T2, FC= 0.29) and subsequent recovery in T3 (FC= 0.44) and T4 (FC= 0.71). (B) hsa-miR-375 had elevated expression level at T3 (FC= 1.78) compared to T1. (C) Expression of hsa-miR-122-5p dropped significantly at T2 (FC= 0.70) with a rebound at T3 (FC= 1.91) and T4 (FC= 2.32). (D) Similarly, hsa-miR-126-5p dropped after conditioning regimen administration (T2, FC=0.74), with a subsequent increase in T3 (FC=1.31). (E) hsa-miR-122-3p had significantly higher expression level at T3 (FC= 4.16) and T4 (FC= 3.03) compared to T1. Asterisks denote the significance level (paired t-test with Bonferroni correction): *- p ≤ 0.05; **- p ≤ 0.01; *** - p ≤ 0.001; **** - p ≤ 0.0001.

**Supplementary Figure 6.** The architecture of the developed neural network MLP-5-7-2.

**Supplementary Figure 7.**  Target prediction analysis of miRNAs included in NN using miRNet. Genes targeted by all three miRNAs in the model are annotated. Overall, three miRNAs- hsa-miR-223-3p, hsa-miR-15b-5p, and hsa-miR-126-5p targeted a total of 3116 genes, and hsa-miR-15b-5p was found to potentially target the highest number of predicted genes (2354 genes), followed by hsa-miR-126-5p (1046 genes) and hsa-miR-223-3p (141 genes). Among targeted genes, 16 were targeted by all three miRNAs, including a component of inhibitor of nuclear factor kappa B kinase complex (CHUK), Interleukin 6 Cytokine Family Signal Transducer (IL6ST), Cell Cycle Associated Protein 1 (CAPRIN1), Mouse double minute 2 homolog (MDM2), specificity protein 1 (SP1), solute carrier family 7 member 5 (SLC7A5).

**Supplementary Figure 8.** KEGG pathway analysis of the miRNAs used in the predictive model for bacteremia.

**Supplementary Figure 9**. Putative tissue sources of miRNAs included in NN identified using DIANA-miTED and miRNATissueAtlas2.

***Supplementary File 2**. LogTPM values of 193 miRNAs included in the analysis from miRNA-seq stage of the study. Absent expression data were filled in with logTPM values of 0.

***Supplementary File 3**. miRNA differential expression analysis results in patients with platelet and neutrophil delayed engraftment (DE).

***Supplementary File 4.** Characteristics of the training, testing and validation subsets.

***Supplementary File 5.** Differential expression analysis of miRNAs in patients with bacteremia in the training set.

***Supplementary File 6.** Prediction tables for training, test and validation sets.

***Supplementary File 7**. miRNA-based neural network source code.

**Supplementary File 1.** Detailed Patients and Methods section.

*Patients and treatment*

The study group consisted of prospectively recruited patients treated with AHSCT at the Department of Hematology and Transplantology, Provincial Multi-Specialized Oncology and Trauma Center, Lodz, Poland. The patients were recruited between February 2015 and January 2022. The flow chart of patient enrollment is presented in Supplementary Figure 1. The study was conducted according to good clinical and laboratory practice rules and the principles of the Declaration of Helsinki. Each patient signed the informed consent for all examinations and procedures. All procedures were approved by the local ethical committee (The Ethical Committee of the Medical University of Lodz, No RNN/424/19/KE). The inclusion criteria were (1) age >18 years; (2) diagnosis of hematologic malignancy; (3) qualification to AHSCT during the treatment schedule. The exclusion criteria included contraindications to AHSCT, mainly Hematopoietic Cell Transplantation-Comorbidity Index (HCT-CI) ≥ 3. The myeloablative conditioning regimen for patients with MM was high-dose melphalan (200 mg/m2), while B(e)EAM was used in lymphoma patients. The latter consisted of carmustine 300 mg/m^2^ or bendamustine 160-200 mg/m^2^ administered at day -6, etoposide 200 mg/m^2,^ and cytarabine 200 mg/m^2^ twice a day given in days -5 to -2 and melphalan 140 mg/m^2^ given at -1 day. All patients have received granulocyte-colony-stimulating factor (G-CSF) from +4 day after AHSCT (72 h after transplantation) until engraftment. Each patient received antimicrobial prophylaxis consisting of ciprofloxacin 500 mg twice daily, acyclovir 800 mg twice daily, and fluconazole 400 mg once daily for patients at high risk for candidiasis.

*Endpoints*

A blood culture was considered positive (bacteremia) when an organism grew in one or more vials, except for coagulase-negative Staphylococci (CoNS), which required two positive blood cultures. Blood samples were collected from the central and peripheral lines of every patient diagnosed with fever. At each site, three blood cultures were conducted, using media for aerobic bacteria, anaerobic bacteria, and fungi. Oral mucositis was classified according to the World Health Organization (WHO) guidelines(1). Neutrophil engraftment was defined as the first three days with an absolute neutrophil count (ANC) >0.5x10^9^/L, and platelet (PLT) engraftment was defined as independence from platelet transfusion for at least seven days with a platelet count of more than >20×10^9^/L (2). There is no consistent definition of ANC/PLT delayed engraftment (DE) in the literature (3), so we used the fourth quartile of duration time to engraftment in patients receiving melphalan (MEL) and B(e)EAM (bendamustine or carmustine, etoposide, cytarabine, melphalan) regimens, respectively.

*miRNA expression analysis*

Serum samples were taken from each patient at four time points: (T1) before conditioning with high-dose chemotherapy, (T2) on the day of AHSCT (day 0), on day +7 (T3), and on +14 day after AHSCT (T4). The blood was centrifuged at 1000x g for 10 min at 4 °C. Serum samples were stored frozen at −80 °C. Patients were divided into two cohorts: in the first, exploratory group (N1=10) serum miRNA-seq was performed, and in the second (N2=69), selected miRNAs were validated using RT-qPCR.

Briefly, total RNA was extracted from 200 μl serum using miRNeasy Serum/Plasma Advanced Kit (QIAGEN) according to the manufacturer's instructions. Sequencing was performed in 40 samples from 10 patients in the exploratory group. Libraries were prepared from 5 μl RNA using QIAseq® miRNA Library Kit following manufacturer recommendations for biofluid samples. Briefly, a preadenylated DNA adapter was ligated to the 3' ends of all miRNAs. Next, an RNA adapter was ligated to the 5' end of mature miRNAs. In the next sequencing step, the RT primer binds to a region of the 3' adapter and facilitates the conversion of the 3'/5' ligated miRNAs into cDNA while assigning a UMI to every miRNA molecule. After reverse transcription, a cleanup of the cDNA was performed using a streamlined magnetic bead-based method. In the library amplification step, the premixed oligos were used in a PCR reaction to assign each sample a unique dual index. Finally, using a streamlined magnetic bead-based method library was cleanup. The concentration of prepared libraries was measured using Qubit 2.0 Fluorometer and Qubit dsDNA HS Assay Kit (both Thermo Fisher Scientific). Library quality was assessed on High Sensitivity D1000 ScreenTape on the Bioanalyzer (both Agilent Technologies) and then pooled in equimolar ratio. Sequencing was performed on a NextSeq 550 System using a single-end read length of 75 nucleotides at an average of 10 million reads per sample (Illumina). In bioinformatics analysis, after adapter cut-off, filtration and mapping, miRNAs were counted based on mapping to reference miRbase 22 (tools: fastp, bowtie, samtools, picard).

The expression levels of selected 21 miRNAs were validated by RT-qPCR using the miRCURY LNA miRNA Custom PCR Panels (QIAGEN) in 69 AHSCT recipients. Briefly, 2 µL of cDNA was synthesized from the obtained total RNA including mature miRNAs (<200 bp), using the miRCURY LNA Reverse Transcription Kit (QIAGEN) according to the manual provided by the manufacturer. Mature miRNAs were polyadenylated by poly(A) polymerase and reverse transcribed into cDNA using oligo-dT primers. The cDNA was stored at −20 °C until further use. Next, a premix of 3 µL of cDNA template (diluted 1:30), 5 µL of 2X miRCURY LNA SYBR Green PCR Master Mix and filled with RNase-free water to the final volume of 10 µL, was aliquoted into the PCR plate. Real-time PCR was performed on a LightCycler480 II Real-Time PCR System (Roche, Pleasanton, CA, USA). Absolute quantification of miRNA was determined using the LightCycler® 480 Software, Version 1.5 (Roche, Mannheim, Germany). Detailed methodology of miRNA-seq and RT-qPCR was described elsewhere (4).

*Sample size estimation*

To assess the sample size needed for our study, we evaluated the diagnostic performance of a standard biomarker used in infectious complications diagnosis- C-reactive protein (CRP). We aimed to develop a classifier capable of predicting infectious complications before their onset, focusing specifically on day 0 during ASCT. At this time point, CRP demonstrated only a modest discriminatory ability, exhibiting an Area Under the Curve (AUC) of 0.59 (95% CI: 0.42-0.77) in identifying patients at risk of bacteremia. We expected that our NN model would reach the AUC threshold of 0.90. Considering a significance level (α) of 0.05 and a statistical power of 0.80, and accounting for a ratio of negative cases to positive cases of 4, our sample size estimation revealed that 35 subjects would be necessary to detect a significant difference in the performance of the two biomarkers based on their ROC curves. Moreover, we expanded our study group accordingly by acknowledging the imperative for model testing and validation and the potential for technical errors resulting in a 10% data loss.

*Statistical analysis*

The expression of miRNAs was calculated according to the ΔCt method (5). NormiRazor was used to measure the stability of miRNAs (18). RT-qPCR data were normalized using an arithmetic average of two miRNAs’ expression levels in a given sample (hsa-miR-27b-3p and hsa-miR-148b-3p) which proved to be the most stable normalization factor. The formula used to calculate the normalized Ct values was:

Normalized ΔCt = (mean Ct of hsa-miR-27b-3p and hsa-miR-148b-3p) - Ct miRNA of interest.

This approach results in higher values for higher miRNA expression enabling straightforward biomarker interpretation. For the visual representation of multidimensional data, heatmap and unsupervised hierarchical clustering were performed.

The miRNAs from miRNA-seq were initially filtered for targets with at least ten counts per million in at least 50% of the samples. Overall, 193 miRNAs were eligible for further analyses (Supplementary Table 1). The expression of miRNAs between consecutive time points was analyzed using repeated measures ANOVA, and miRNAs with significant differential expression in pairwise comparisons between all four time points (paired t-test with Bonferroni correction) were selected for further experiments.

Ten samples (3.6%) out of 276 evaluated in RT-qPCR stage of this study stage were omitted because they did not meet quality control criteria, resulting in excluding two complete series (T1-T4) and two additional samples (one at T3 and one at T4). Differential expression analysis was performed using an unequal variances t-test. The target genes of miRNAs included in the neural network construction were predicted using miRNet 2.0 (http://www.mirnet.ca/ accessed 26.04.2023) (6). Using the miRNet, an enrichment analysis of Kyoto Encyclopedia of Genes and Genomes (KEGG) pathways was conducted to investigate the role of selected miRNAs in biological processes and signaling pathways.

The classification model was based on artificial neural networks, which were previously shown to be a successful approach for model development of miRNA biomarkers (4). We built 200000 neural networks using a split set approach and retained the best in properly assigning cases to classes in the hold-out validation subset. The networks were built in a semi-automated way through a grid search procedure. All statistical analyses were conducted using Statistica Version 13.1 (TIBCO, Palo Alto, CA, USA) and R programming language (version 4.0.2) with the OmicSelector R package (7). The potential tissue sources of miRNAs included in NN were predicted using DIANA-miTED and miRNATissueAtlas2 (8,9).

**References:**

1. Niscola P, Romani C, Cupelli L, Scaramucci L, Tendas A, Dentamaro T, et al. Mucositis in patients with hematologic malignancies: an overview. Haematologica. 2007 Feb;92(2):222–31.

2. Hutt D. Engraftment, Graft Failure, and Rejection. In: Kenyon M, Babic A, editors. Cham (CH); 2018. p. 259–70.

3. Lutfi F, Skelton IV WP, Wang Y, Rosenau E, Farhadfar N, Murthy H, et al. Clinical predictors of delayed engraftment in autologous hematopoietic cell transplant recipients. Hematol Oncol Stem Cell Ther. 2020;13(1):23–31.

4. Elias KM, Fendler W, Stawiski K, Fiascone SJ, Vitonis AF, Berkowitz RS, et al. Diagnostic potential for a serum miRNA neural network for detection of ovarian cancer. Sawyers CL, editor. Elife. 2017;6:e28932.

5. Livak KJ, Schmittgen TD. Analysis of Relative Gene Expression Data Using Real-Time Quantitative PCR and the 2−ΔΔCT Method. Methods. 2001;25(4):402–8.

6. Chang L, Zhou G, Soufan O, Xia J. miRNet 2.0: network-based visual analytics for miRNA functional analysis and systems biology. Nucleic Acids Res. 2020 Jul 2;48(W1):W244–51.

7. Stawiski K, Kaszkowiak M, Mikulski D, Hogendorf P, Durczyński A, Strzelczyk J, et al. OmicSelector: automatic feature selection and deep learning modeling for omic experiments. bioRxiv. 2022 Jan 1;2022.06.01.494299.

8. Keller A, Gröger L, Tschernig T, Solomon J, Laham O, Schaum N, et al. miRNATissueAtlas2: an update to the human miRNA tissue atlas. Nucleic Acids Res [Internet]. 2022 Jan 7;50(D1):D211–21. Available from: https://doi.org/10.1093/nar/gkab808

9. Kavakiotis I, Alexiou A, Tastsoglou S, Vlachos IS, Hatzigeorgiou AG. DIANA-miTED: a microRNA tissue expression database. Nucleic Acids Res [Internet]. 2022 Jan 7;50(D1):D1055–61. Available from: https://doi.org/10.1093/nar/gkab733

**Supplementary Table 1.** Clinical characteristics of patients included into the study.

| **miRNA-seq group (N=10)** | | | |
| --- | --- | --- | --- |
| **Variable** | **N (%)** | | |
| Disease | MM: 4 (40.0)  HL: 3 (30.0)  MCL: 1 (10.0)  DLBCL: 1 (10.0)  AITL: 1 (10.0) | | |
| Age at AHSCT  Median (27-75%), years | 45.0 (40.0-53.5) | | |
| Sex | F: 6 (60.0)  M: 4 (40.0) | | |
| Conditioning regimen | Mel-200: 4 (40.0)  BeEAM: 5 (50.0)  BEAM: 1 (10.0) | | |
| Number of CD34+ cells transplanted  (× 10^6^ cells/kg)  Median (27-75%) | 4.34 (3.31-5.69) | | |
| Mucositis- any grade | 8 (80.0) | | |
| Mucositis – grade ≥2 | 7 (70.0) | | |
| Bacteremia | 2 (20.0) | | |
| Neutropenic fever | 5 (50.0) | | |
| Days to PLT engraftment  Median (27-75%) | 20.0 (13.0-24.0) | | |
| Days to ANC engraftment  Median (27-75%) | 10 (10.0-11.0) | | |
| **RT-qPCR group (N=69)*** | | | |
| **Variable** | **N (%)** | | |
| Disease | MM: 51 (73.9)  HL: 9 (13.0)  MCL:4 (5.8)  DLBCL: 2 (2.9)  Other: 3 (4.3) | | |
| Conditioning regimen | Mel-200: 36 (52.2)  Mel-140: 11 (15.9)  Mel-100: 5 (7.2)  BeEAM: 13 (24.6)  BEAM: 4 (5.8) | | |
| Variable | MM | ML | *p* |
| Sex | F: 27 (52.9)  M: 24 (47.1) | 8 (44.4)  10 (55.6) | 0.7296 |
| Age at AHSCT  Median (27-75%), years | 59.4 (55.1-66.1) | 45.6 (39.5-53.0) | <0.0001 |
| Number of CD34+ cells transplanted  (× 10^6^ cells/kg)  Median (27-75%) | 3.9 (3.0-5.3) | 4.4 (3.1-5.7) | 0.5237 |
| Mucositis- any grade | 34 (66.7) | 17 (94.4) | 0.0275 |
| Mucositis – grade ≥2 | 18 (36.3) | 16 (88.9) | <0.0001 |
| Bacteremia | 10 (19.6) | 7 (38.9) | 0.1583 |
| Neutropenic fever | 31 (60.8) | 15 (83.3) | 0.1459 |
| Days to PLT engraftment  Median (27-75%) | 16 (13-19) | 20.5 (17-25) | <0.0044 |
| Days to ANC engraftment  Median (27-75%) | 11 (11-12) | 10.5 (10-11) | 0.0323 |

AITL- Angioimmunoblastic T-cell lymphoma; ANC- absolute neutrophil count; BEAM- carmustine , etoposide, cytarabine, melphalan; BeEAM- bendamustine, etoposide, cytarabine, melphalan; DLBCL- diffuse large B-cell lymphoma; HL- Hodgkin Lymphoma; 27-75%- interquartile range; MCL- mantle cell lymphoma; MEL- melphalan; ML- malignant lymphoma; MM- multiple myeloma; PLT- platelets

*samples from 2 patients collected at timepoint 1 and 2 respectively, were excluded from the analysis due to considerable hemolysis. As samples were collected in sequence, all qPCR data from both patients was thus impossible to use in further analyses.

**Supplementary Figure 1**. The graphical representation of the study protocol.


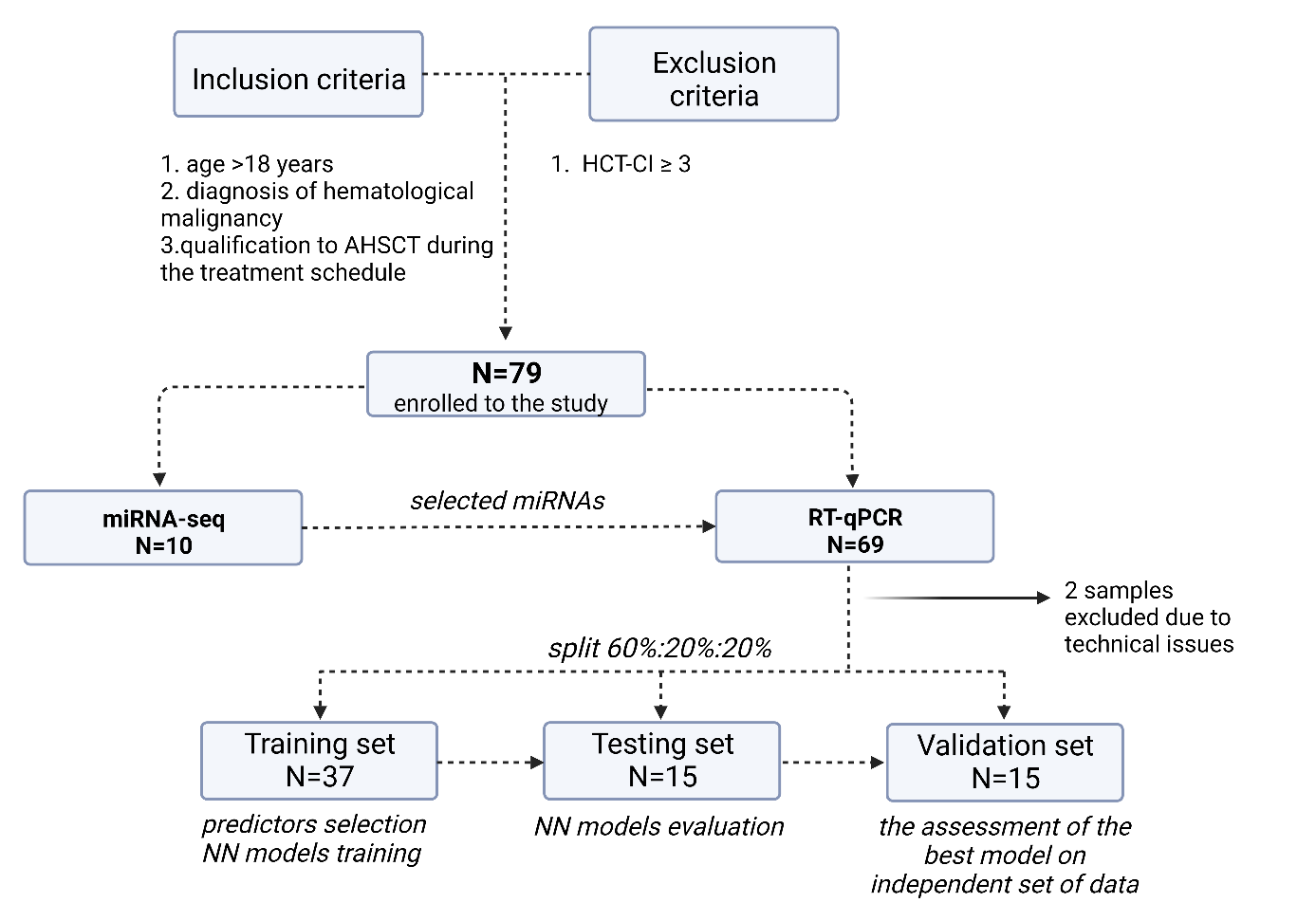


**Supplementary Figure 2.** miRNA expression from miRNA-seq stage of the study- volcano plots demonstrating pairwise comparisons between study timepoints. A. Paired comparison- T1 vs T2, none of miRNAs remained statistically significant after false discovery rate (FDR) correction. B. T1 vs T3- four miRNAs were significantly upregulated- hsa-miR-320b (fold change, FC=3.1, p=0.007); hsa-miR-320c (FC= 3.9, p=0.007), hsa-miR-320a-3p (FC=2.0, p=0.009) and hsa-miR-320d (FC=3.9, p=0.042) and one miRNA was significantly downregulated- hsa-miR-223-3p (FC=0.3, p=0.048). C. T1 vs T4- three miRNAs were significantly downregulated: hsa-let-7f-5p (FC=0.4, p=0.004), hsa-let-7i-5p (FC=0.6, p=0.010), hsa-miR-155-5p (FC=0.4, p=0.019); one miRNA was significantly upregulated- hsa-miR-320c (FC= 2.4, p=0.049). D. T2 vs T3- two miRNAs were significantly downregulated- hsa-miR-18a-5p (FC=0.2, p=0.035) and hsa-miR-223-3p (FC=0.4, p=0.033). E. T3 vs T4- two miRNAs were significantly deregulated- hsa-miR-486-5p (FC=0.7, p=0.024) and hsa-miR-96-5p (FC=8.3, p=0.036). F. T2 vs T4- none of miRNAs remained statistically significant after FDR correction.

**
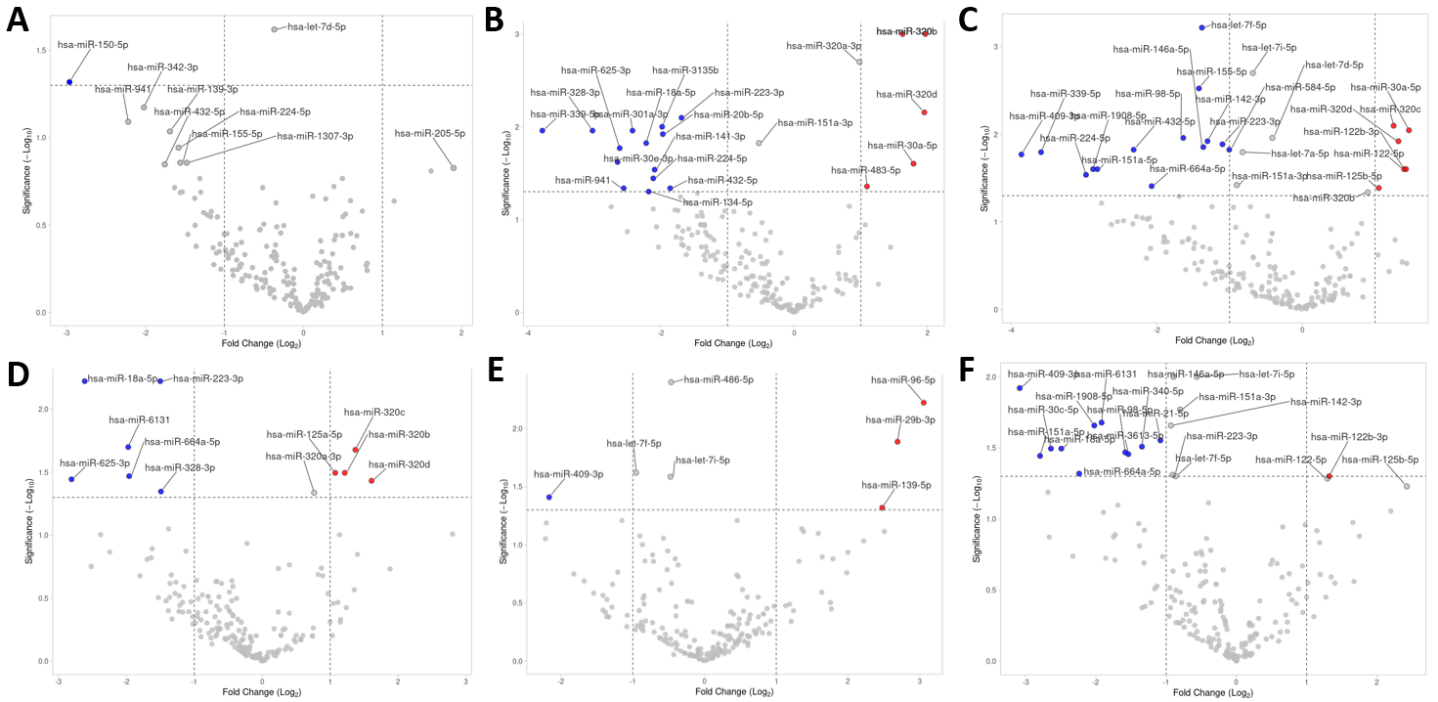
**

**Supplementary Figure 3.** Correlations between time to engraftment and expression level of miRNAs measured at baseline (timepoint 1) in the miRNA-seq stage of the study. In total 5 miRNAs that showed significant correlations with both neutrophil and platelet engraftment times were subsequently selected for qPCR validation. (A, B) hsa-miR-125a-5p; (C, D) hsa-miR-99b-5p; (E, F) hsa-miR-361-3p; (G, H) hsa-miR-486-3p; (I, J) hsa-miR-92b-3p.

**
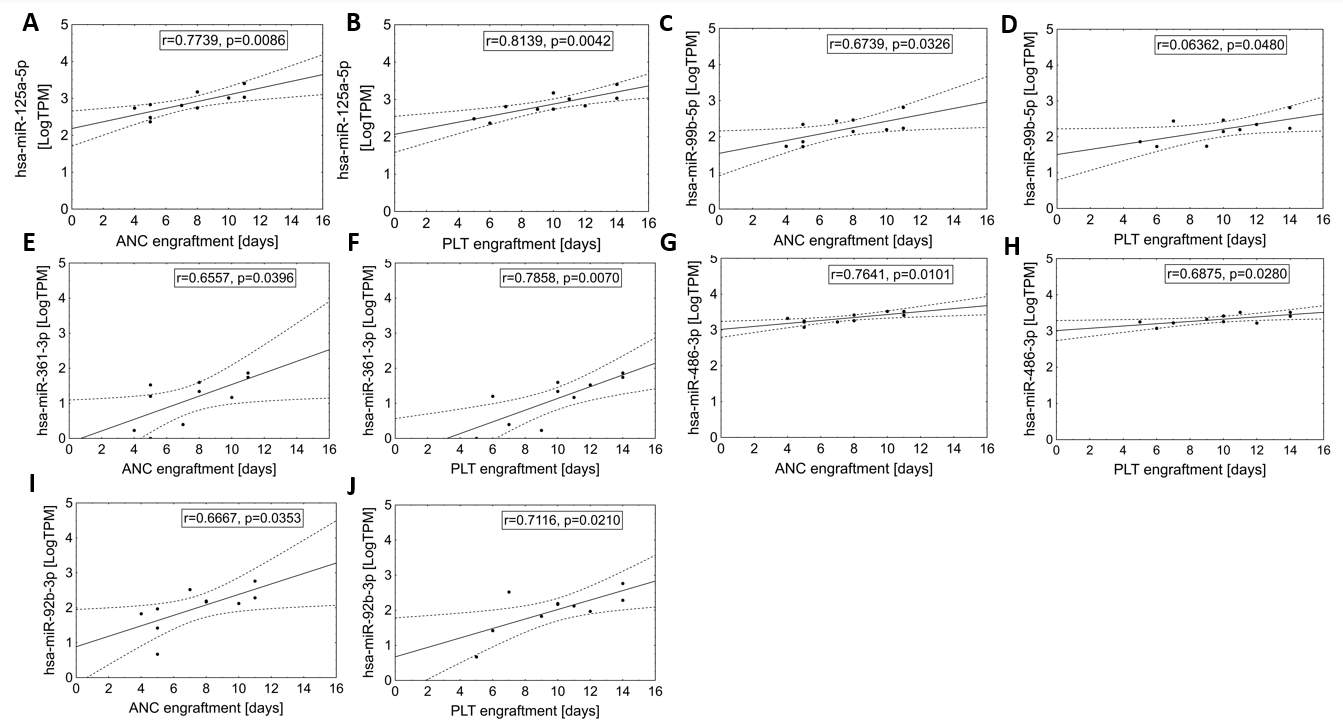
**

**Supplementary Figure 4.** Validation of sequencing data using RT-qPCR. Comparisons of log2FC values between miRNA-seq and RT-qPCR in subsequent timepoints: A. T1 vs T2; B. T1 vs T3; C. T1 vs. T4.

*
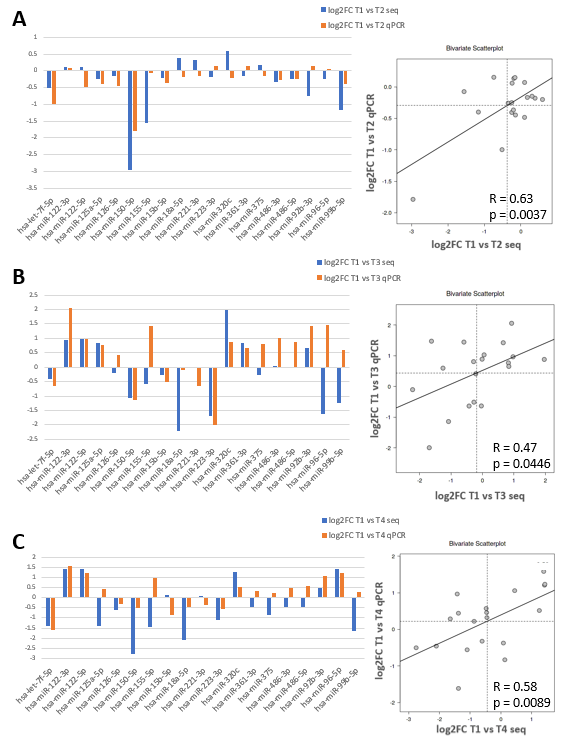
*

**Supplementary Figure 5.** Changes in serum expression of miRNAs related to radiotherapy response. (A) hsa-miR-150-5p had the highest expression at T1, with the drop after administration of conditioning chemotherapy (T2, FC= 0.29) and subsequent recovery in T3 (FC= 0.44) and T4 (FC= 0.71). (B) hsa-miR-375 had elevated expression level at T3 (FC= 1.78) compared to T1. (C) Expression of hsa-miR-122-5p dropped significantly at T2 (FC= 0.70) with a rebound at T3 (FC= 1.91) and T4 (FC= 2.32). (D) Similarly, hsa-miR-126-5p dropped after conditioning regimen administration (T2, FC=0.74), with a subsequent increase in T3 (FC=1.31). (E) hsa-miR-122-3p had significantly higher expression level at T3 (FC= 4.16) and T4 (FC= 3.03) compared to T1. Asterisks denote the significance level (paired t-test with Bonferroni correction): *- p ≤ 0.05; **- p ≤ 0.01; *** - p ≤ 0.001; **** - p ≤ 0.0001.

*
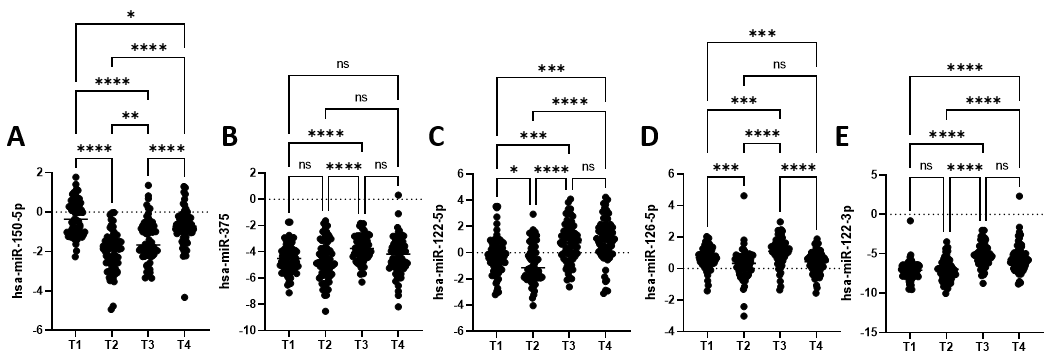
*

**Supplementary Figure 6.** Architecture of the developed neural network MLP-5-7-2.

**
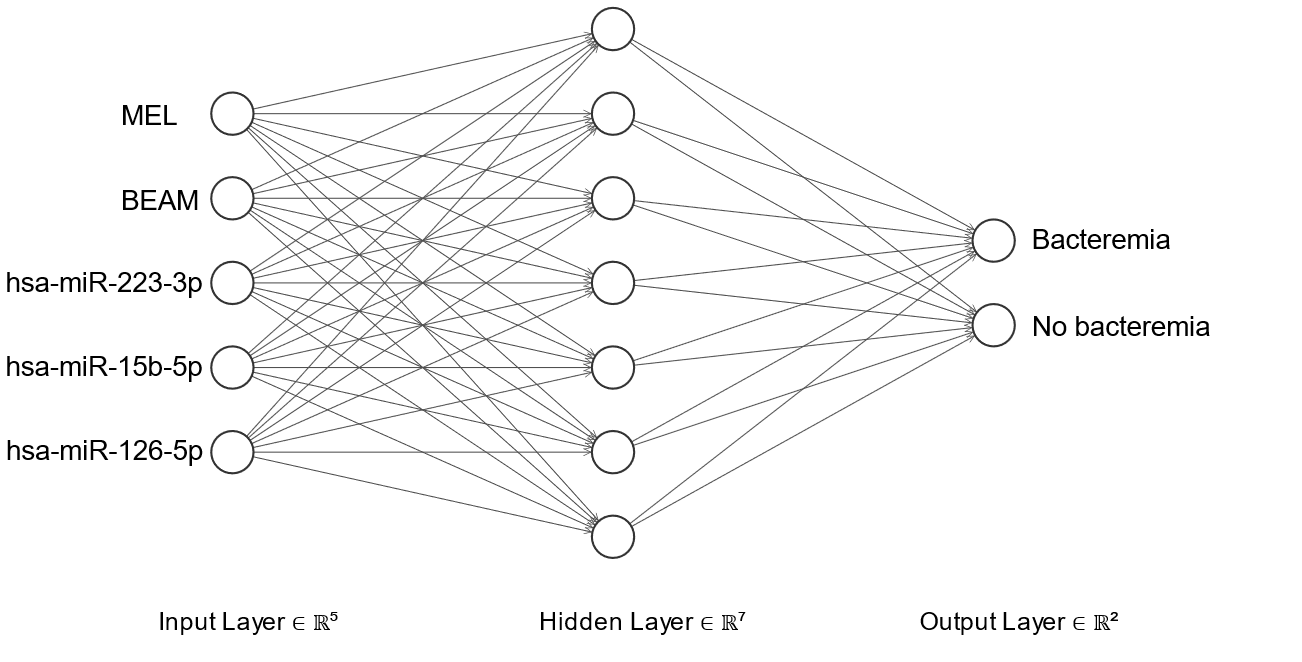
**

**Supplementary Figure 7.**  Target prediction analysis of miRNAs included in NN using miRNet. Genes targeted by all three miRNAs in the model are annotated. Overall, three miRNAs- hsa-miR-223-3p, hsa-miR-15b-5p, and hsa-miR-126-5p targeted total of 3116 genes, and hsa-miR-15b-5p was found to potentially target the highest number of predicted genes (2354 genes), followed by hsa-miR-126-5p (1046 genes) and hsa-miR-223-3p (141 genes). Among targeted genes, 16 were targeted by all three miRNAs, including a component of inhibitor of nuclear factor kappa B kinase complex (CHUK), Interleukin 6 Cytokine Family Signal Transducer (IL6ST), Cell Cycle Associated Protein 1 (CAPRIN1), Mouse double minute 2 homolog (MDM2), specificity protein 1 (SP1), solute carrier family 7 member 5 (SLC7A5).

*
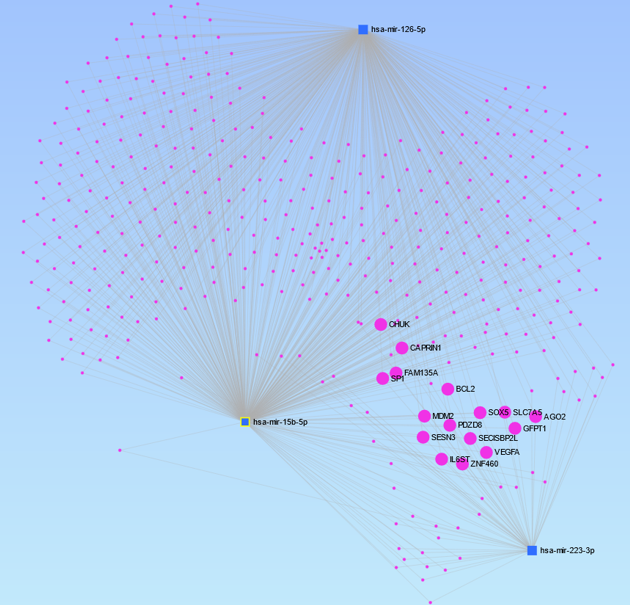
*

**Supplementary Figure 8.** KEGG pathway analysis of the miRNAs used in the predictive model for bacteremia.

*
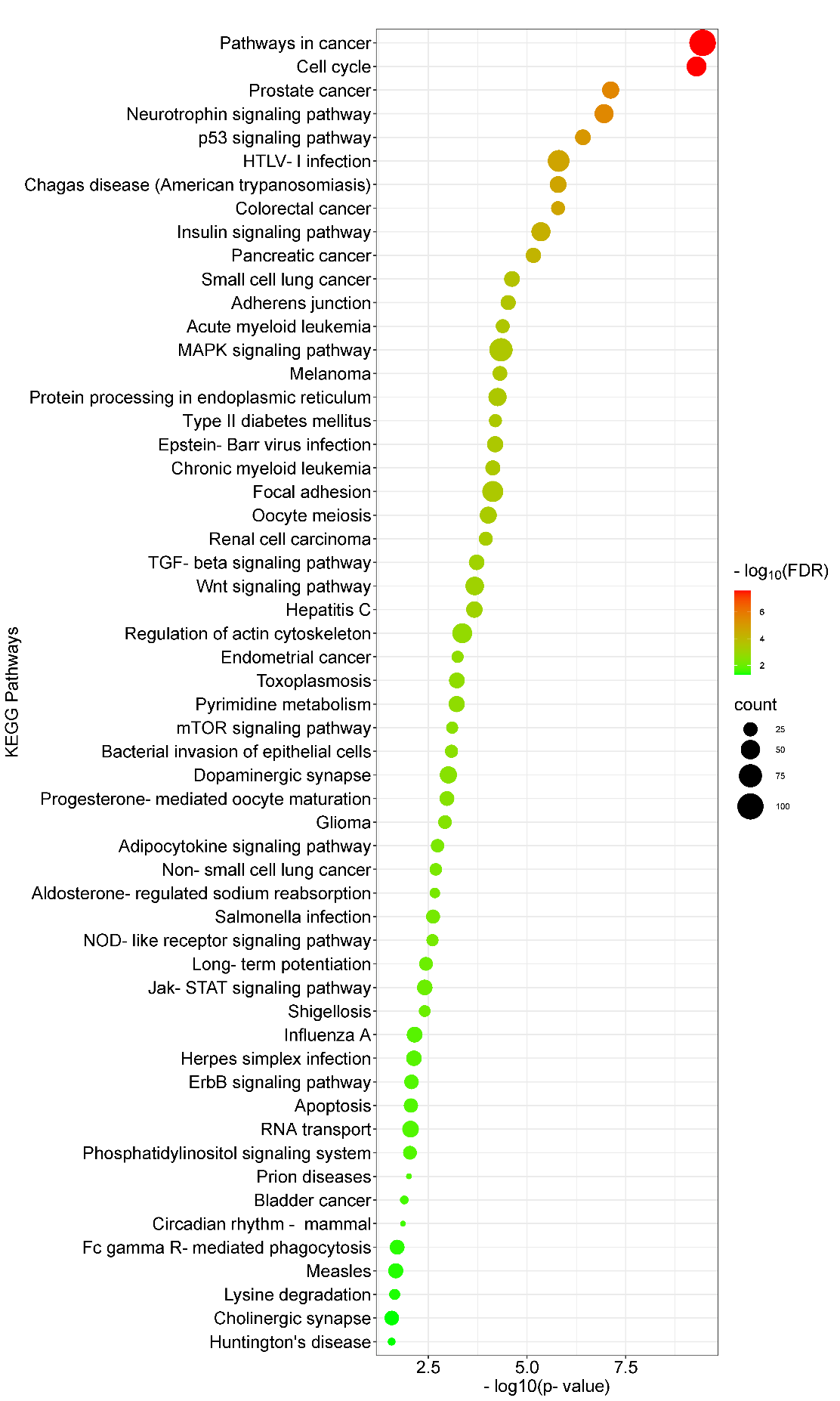
*

**Supplementary Figure 9**. Potential tissue sources of miRNAs included in NN predicted using DIANA-miTED and miRNATissueAtlas2.

*
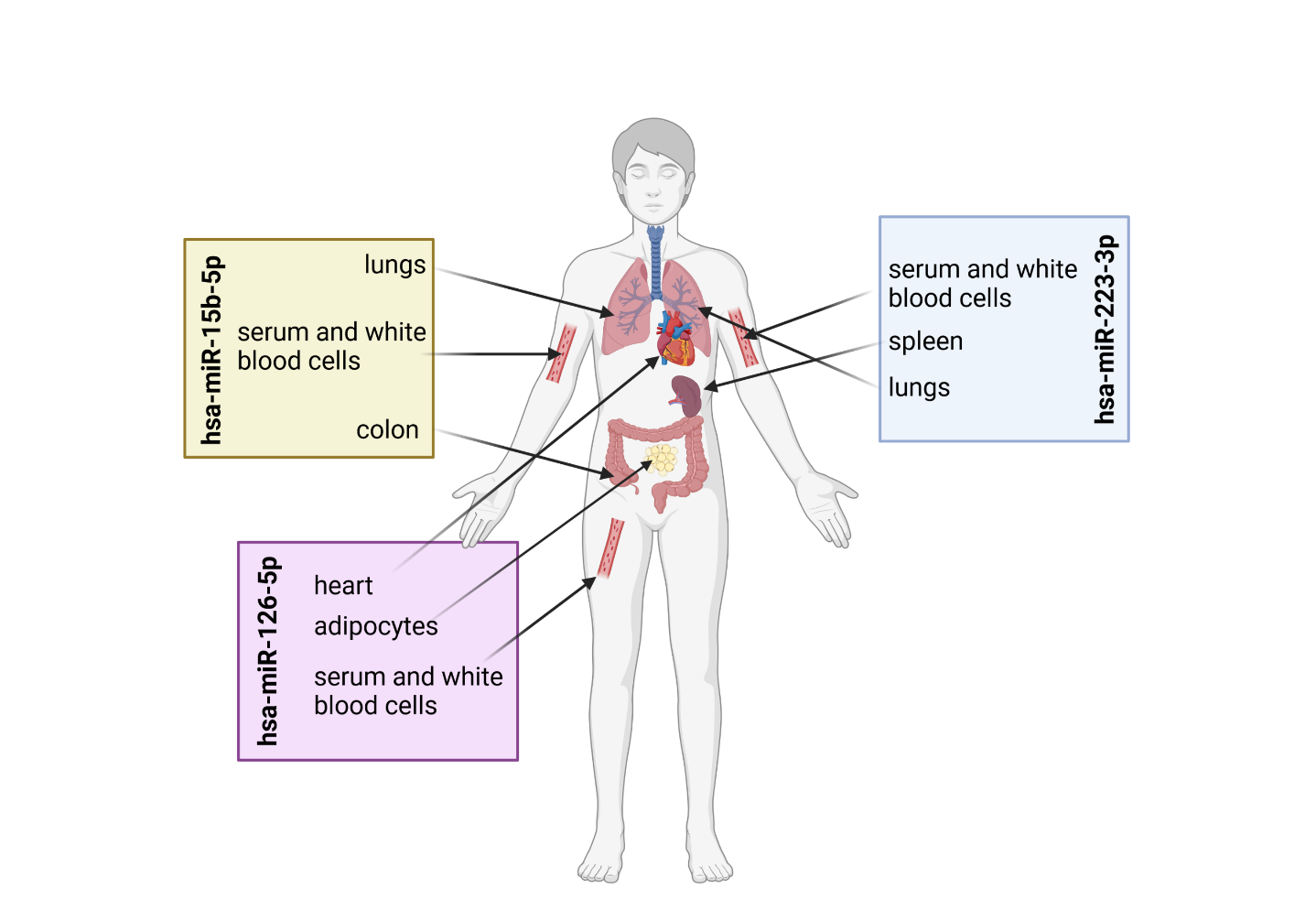
*
